# The Medical Journal Trust and the Independent Medical Press

**Published:** 1905-07

**Authors:** 


### THE MEDICAL JOURNAL TRUST AND THE INDEPENDENT MEDICAL PRESS.

*Gentlemen of the Association of American Medical Editors:**

The organization of the medical profession of America upon a plan that would secure unity and cohesion has long been recognized as a necessity in order to enable them to exert influence and secure medical and sanitary legislation in the interest of the public health. To this end various schemes have been tried in the several

*This was read at the Portland meeting of medical editors.

States, all, or most of which, have proved a failure. The medical press of the country with one accord has co-operated in the efforts to secure from Congress and from the several State Legislatures laws for the enforcement of preventive measures against disease,—to protect the public from quacks,—to provide for pure food, drugs, drinks, etc.,—but every effort has been a failure. The medical profession has not been able to impress Congress and the State Legislatures with the necessity for such legislation, and everything that has been asked for has been refused, and—in Texas—laughed at. In Texas it is a felony to procure anatomical material for teaching anatomy in our great school—hence grave robbing, for the material must be forthcoming. The State Medical Association of Texas, at the request of the faculty of the Medical Department of the State University, asked the Legislature to legalize dissection and to give to the college the unclaimed cadavers from our charity institutions. The request was ridiculed and laughed out of court. The request of the American Medical Association that a physician be put upon the Panama Canal Commission was refused, as has been the request for a pure food bill and for a National Board of Health.

It was thought that when organized from the ground up the profession, speaking as one man, could command attention. We are now organized—and still we can not do anything.

Organization for a legitimate purpose is most desirable, but when it is used for selfish, pecuniary or political purposes it is a power for evil, and is detrimental to the best interests of the profession.

When the plan of organization now in operation in nearly all the States was proposed, it was hailed with loud acclaim and rejoicing, and the medical press "whooped 'em up" with a vim. The journals and individual physicians co-operated with the *Journal of the American Medical Association's paid organizer*, and in every State organization has been effected, and never a suspicion had we of the ulterior and nefarious object to be accomplished by the originators of the scheme—*The Journal of the American Medical Association*. But in light of developments the independent medical press can now see that they have cut the ground from under their feet, tied their hands, and been hoisted on their own petard. WHY should the *Journal of the American Medical Association* pay large sums of money to secure the organization of State medical associations? In light of developments it is clearly seen that it was an *investment*, which is already paying large returns. After whipping into the county societies every man who could be coaxed or driven into the organization, and through the county society into the State Association, and through the State Association into the American Medical Association—where he becomes a member by virtue of his membership of his State Association, he pays $5 and gets the great organ of the National Association, a weekly. It is to be put into the hands of practically every doctor in every State. It is sent him free, as a bonus for his membership—hence every new members means $5 added to the coffer (already overflowing) of the great *Journal A. M. A.* There is the bug.

But that is not all. To facilitate this process, every State Association is induced to establish its own journal—ostensibly to publish the proceedings of the Association, but practically for political purposes, and they become whippers in and drummers for the *Journal A. M. A.;* feeders to the rapacious maw of the central power—the *Octopus* at Chicago. Thus a great Medical Journal Trust has been organized.

These State Association journals are *sent free* to every member in consideration of his annual dues (in Texas $2),—and every member of every county society in every State is expected to pay the fee, or the councilors will make it hot for him. The means of applying the pressure consists of giving it out that every reputable physician is a member of his county society; if not, the people will want to know why, and he is to be boycotted by them and by corporations, insurance companies, etc., and by threats of publishing a list of "who's who"—so that it is made to appear that not to be in "who's who," and not inside of the corral, is to be "not in good standing." Already, in Texas, the Secretary of the State Medical Association (who is to edit the *State Association Journal*) publishes the statement that about 50 per cent of the "regular" physicians are enrolled and this, he says, is 80 to 90 per cent of the progressive and educated physicians of the State. The inference is clear. Those not "in it" are not "progressive and educated."

Such methods are reprehensible and smack of trades unionism.

Thus it will be seen that every physician in every State who is entitled to membership is to be driven into the fold *nolens volens*—or take the consequences; and thus it will be seen that every physician in every county in every State is to be furnished free of charge (in consideration of membership fee) the big weekly and his State monthly.

Where, then, comes in the personally owned and independent medical journals? Where will their readers come from? The great Medical Trust thus established furnishes your readers with all they need in the way of journal literature and society matters,—and the independent journals having been made a means to this end, stepping stones to the summit of power which is to be further used for oppression, coercion and profit, are kicked out and ignored,—their past services forgotten.

Nor is this all. The *Journal A. M. A.*, and the State journals, so called,—using membership money for the publication of the periodicals,—take such advertisements as are carried by the independent journals at *less rates* than these publications can afford to carry them, and thus you gentlemen of the independent press find your occupation gone, your revenues cut off and diverted into the coffers of the organization you have been mainly instrumental in creating.

What are you going to do about it?

This scheme, practically successful even now, through the influence of the independent press, is a menace to the very existence of an independent medical press. It is not to the interest of the organizers of the scheme that an independent press should exist. They dare criticise the great I AM, and must be suppressed. The policy inaugurated and carried out by that journal is biased by the personal likes and dislikes of the management. In collating the current medical literature each week the managing editor never finds anything worth mentioning in the journals that have dared to raise their voices against it. Ask Register, ask Lanphear, ask the *New York Medical Journal.* This policy I am sure does not reflect the views of the great majority of American physicians, of whom about 85 per cent have not been corralled, nor of the 15 per cent who are members of the A. M. A. In support of this assertion I submit the following:

The American Congress on Tuberculosis was the first body organized to fight tuberculosis. It was organized in 1900, and held its fourth annual session in St. Louis in October, 1904, under the auspices and upon invitation of the World's Fair management, and the patronage and authority of the Federal government. Secretary of State Hay invited delegates to be sent from all foreign countries. The meeting was successful, largely attended, and gave the first impetus to the movement now so general, enlisting influence that will be effective in limiting the spread of consumption. It was participated in by many of the most distinguished physicians from Canada and Nova Scotia, Mexico, Central America, South America, Cuba and Porto Rico and of the South and West; Illinois, the home of the Octopus, being largely represented by prominent members of the A. M. A. and readers of the great

Octopus. *Dr. Simmons, ye editor, devoted one inch of the valuable space at his disposal to a sneer at the "so-called Congress on Tuberculosis."* Common decency, or, at least, a decent regard for his readers, many of whom are members of the Congress, working earnestly in the cause of humanity and the public health, demanded the support and approval of the great so-called "representative organ" of the great A. M. A., and the neglect of it was so clearly the result of personal bias and prejudice towards the organizers of the Congress (the Medico-Legal Society) on the part of ye narrow editor as to excite disgust.

This subject, gentlemen, in my deliberate judgment, demands your serious consideration. I have no remedy to suggest, unless it be a change of management, editorial and business, of the *Journal of the American Medical Association.* It is for you in your organized capacity to make your voices heard, and your influence felt for a change of policy which is so arbitrary and unjust and so ruinous to your influence and interests.

I regret my inability to be with you.

